# Supplementary material for: Structural basis of apoptosis induction by the mitochondrial voltage-dependent anion channel
Source: Nat Commun. 2025 Oct 27;16:9481. doi: 10.1038/s41467-025-65363-1 (PMC12559223; doi:10.1038/s41467-025-65363-1)
Supplement: Supplementary file 1 — Supplementary Information [file 41467_2025_65363_MOESM1_ESM.pdf]

Structural basis of apoptosis induction by the mitochondrial voltage dependent anion channel

Supplementary Tables

Supplementary Table 1: Cryo-EM data collection statistics

	cMSP1D1-VDAC1 monomer (EMD-55061)	cMSP1D1-VDAC1 dimer (EMD-55062)	cMSP1ΔH5-VDAC1 monomer (EMD-55094)
**Data collection and processing**			
Magnification	270,000	270,000	270,000
Voltage (kV)	300	300	300
Electron exposure (e–/Å^2)	60	60	50
Defocus range (μm)	0.8-2.0	0.8-2.0	0.4-2.0
Pixel size (Å)	0.46	0.46	0.46
Symmetry imposed	C1	C1	C1
Movies (no.)	10,258	12,000	20,380
Initial particle images (no.)	1,800,035	179,411	4,011,760
Final particle images (no.)	169,625	31,802	74,197
Map resolution (Å)	7.21	6.99	5.73
FSC threshold	0.143	0.143	0.143
Map resolution range (Å)	2.50-7.80	3.19-7.72	5.06-9.32

Supplementary Table 2: MD simulation setup parameters.

**Parameter**	**VDAC1 helix inside**	**VDAC1 helix outside**	**VDAC1-BclxL complex**
Simulation time (µs)	5	5	0.2
Timestep (fs)	2	2	2
Lipids per leaflet	151 POPC, 30 POPG	120 POPC, 30 POPG	70 DMPC
Temperature (K)	310.15	310.15	310.15
Pressure (bar)	1	1	1
Unit cell shape and size (Å)	Cubic: 144 x 144 x 96	Cubic: 120 x 120 x 108	Hexagonal: 86 x 43;75 x 120
Total number of atoms	125291	105325	71223
Number of water molecules	22530	20354	16030
Salt concentration (KCl)	150 mM	150 mM	150 mM
Simulation mode	NPT	NPT	NPT
Software	Gromacs 2025.2 CUDA	Gromacs 2025.2 CUDA	NAMD v2.12

**Equilibration**						
Force constants	Cycle 1 (125 ps, 1 fs timestep)	Cycle 2 (125 ps, 1 fs timestep)	Cycle 3 (125 ps, 1 fs timestep)	Cycle 4 (500 ps, 2 fs timestep)	Cycle 5 (500 ps, 2 fs timestep)	Cycle 6 (500 ps, 2 fs timestep)
Protein backbone	10.0	5.0	2.5	1.0	0.5	0.1
Protein side chains	5.0	2.5	1.0	0.5	0.1	0.0
Water	2.5	2.5	1.0	0.5	0.1	0.0
Lipid tails	2.5	2.5	1.0	0.5	0.1	0.0
Lipid head groups	2.5	2.5	1.0	0.5	0.1	0.0
Ions	10.0	0.0	0.0	0.0	0.0	0.0

Supplementary Table 3. Data collection and refinement statistics for BclxL in complex with VDAC1-N

	BclxL-VDAC-N complex
**Data collection**	
Space group	*C*222$_1$
Cell dimensions	
a, *b*, *c* (Å)	35.12, 98.75, 103.91
α, β, γ (°)	90.00, 90.00, 90.00
Resolution (Å)	50–1.95 (2.00–1.95)*
R_{merge}	10.0 (64.6)
$I / \sigma I$	12.6 (2.3)
Completeness (%)	98.7 (96.2)
Redundancy	4.1 (3.5)
**Refinement**	
Resolution (Å)	50–1.95 (2.00–1.95)
No. reflections	12,735
R_{work} / R_{free}	16.98 / 20.06
No. atoms	
Protein	1,206
Peptide	136
Ion	15
Water	80
B-factors	
Protein	35.7
Peptide	54.8
Ion	49.9
Water	42.5
R.m.s. deviations	
Bond lengths (Å)	0.008
Bond angles (°)	1.76

*Only one crystal was used for the data collection.
Values in parentheses are for highest-resolution shell.

Supplementary Table 4: Alanine scan of the linear peptide derived from VDAC1-N.

Sequence (Y7 to G21)	*m/z*, [M+H$^+$] (calc)	*m/z*, [M+H$^+$] (found)	Purity (UV)
Y A D L G K S A R D V F T K G	1627.8	1627.9	97%
A A D L G K S A R D V F T K G	1536.8	1536.8	>98%
Y A A L G K S A R D V F T K G	1583.7	1583.9	>98%
Y A D A G K S A R D V F T K G	1585.8	1585.6	>98%
Y A D L A K S A R D V F T K G	1641.9	1641.8	>98%
Y A D L G A S A R D V F T K G*	1570.8	1571.1	95%
Y A D L G K A A R D V F T K G	1611.8	1611.7	>98%
Y A D L G K S A A D V F T K G*	1542.8	1542.7	95%
Y A D L G K S A R A V F T K G	1583.9	1583.9	>98%
Y A D L G K S A R D A F T K G	1599.8	1599.7	98%
Y A D L G K S A R D V A T K G	1551.8	1551.9	96%
Y A D L G K S A R D V F A K G	1597.8	1597.7	98%

* The underlined peptides (K12A and R15A) could not be used in binding experiments due to insolubility in aqueous buffer (no affinity data in Fig. 4f and Supporting Fig. 11b).

Supplementary Table 5: DNA primers used for construct design and mutagenesis

Name	Sequence (5' to 3')*
VDAC1-V3M-fw	GTTTAACTTTAAGAAGGAGATATACATATGGCTATGCCACCC ACGTATGCCGATC
VDAC1-V3M-rev	GATCGGCATACGTGGGTGGCATAGCCATATGTATATCTCCTT CTTAAAGTTAAAC
VDAC1-T6C-fw	ATGGCTGTGCCACCCTGTTATGCCGATCTTGGCAAATC
VDAC1-T6C-rev	GATTTGCCAAGATCGGCATAACAGGGTGGCACAGCCAT
VDAC1-L10C-fw	CACCCACGTATGCCGATTGTGGCAAATCTGCCAGGGATG
VDAC1-L10C-rev	CATCCCTGGCAGATTTGCCACAATCGGCATACGTGGGTG
VDAC1-E73V-fw	CGGCCTGACGTTTACAGTGAAATGGAATACCGACA
VDAC1-E73V-rev	TGTCGGTATTCCATTTCACTGTAAACGTCAGGCCG
VDAC1-C172A-fw	GCACATTAACCTGGGCGCAGACATGGATTTCGACATTG
VDAC1-C172A-rev	CAATGTCGAAATCCATGTCTGCGCCCAGGTTAATGTGC
VDAC1-C232S-fw	CAGATTGACCCTGACGCCAGCTTCTCGGCTAAAGTGAACAAC
VDAC1-C232S-rev	GTTGTTCACTTTAGCCGAGAAGCTGGCGTCAGGGTCAATCTG
VDAC-N(1-26)-into-BclxL-fw	GGGCCAGGAACGCCTCGAGGGTGGTATGGCTGTGCCACCCAC GTATG
VDAC-N(1-26)-into-BclxL-rev	GGGCTATGGATTTGGCTTAGGCGGTCACCACCACCACCACCA CTGAG
VDAC1-N(2-25) into pET-GB1-fw	GGAGATATAGCTAGCATGGCTGTGCCACCCACGTATGCC
VDAC1-N(2-25) into pET-GB1-rev	CACCAAGGGCTATGGATTTGGCTTATAATGACTCGAGTATAT CTCC
VDAC2-N(2-36) into pET-GB1-fw	CTTCACGGTAACCGAAATCCCAACGACCATGGCGACCCATGG CCAGAC
VDAC2-N(2-36) into pET-GB1-rev	CGGTGGCAGCAGCCAACTCAGCCAAAGCCAAAGCCTTTGTTA AAAATATCGC

* Mutagenesis site is underlined.

Supplementary Figures

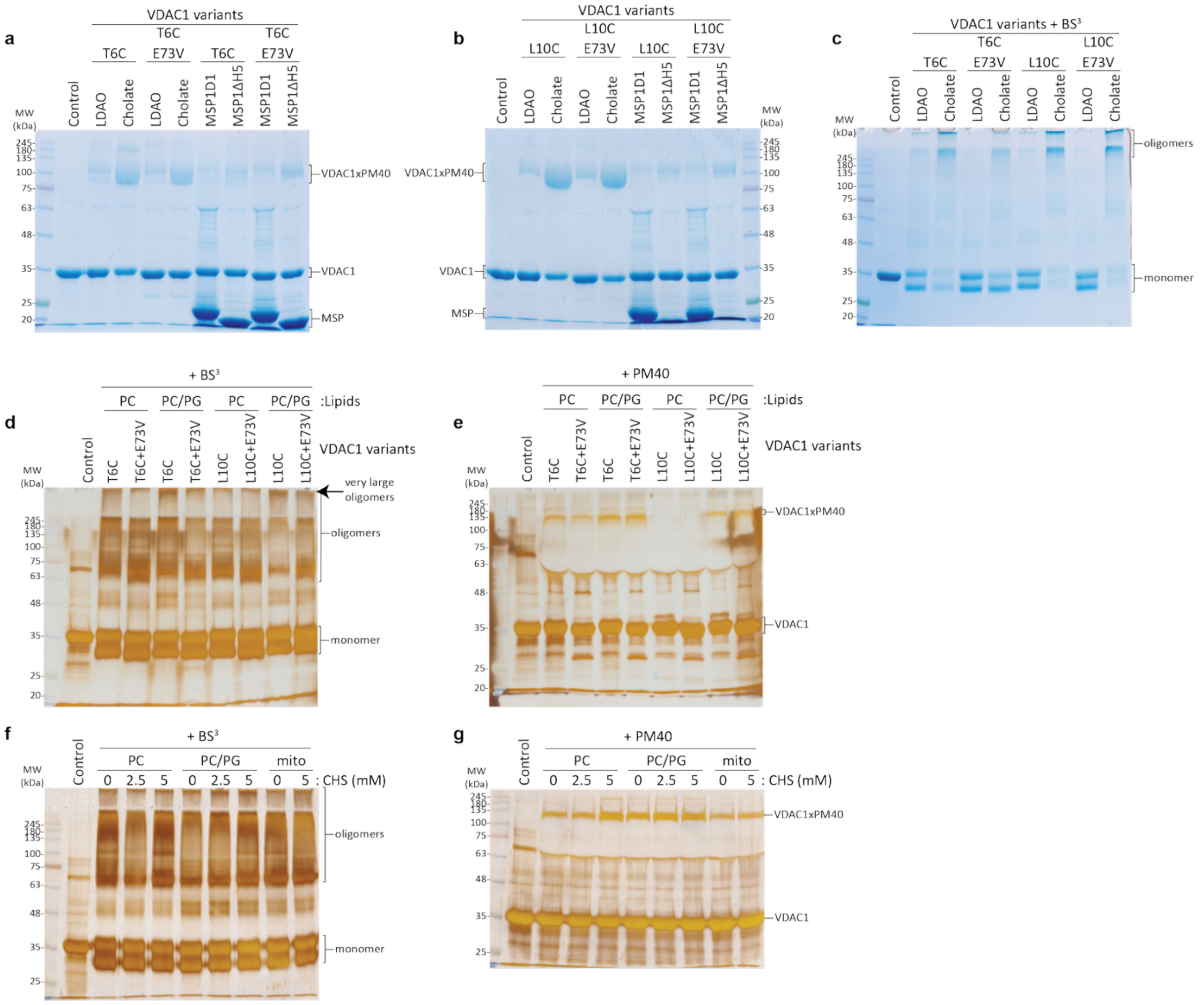

Supplementary Figure 1. **Chemical crosslinking and modification to probe VDAC1 oligomerization and α-helix exposure. a** Chemical modification of VDAC1-T6C with polyethyleneglycol (PEG)-maleimide of 40 kDa (PM40) in different detergents and lipid nanodiscs. The VDAC1 point mutation E73V, that was reported to show a tighter interaction between the α-helix and the β-barrel wall[1], was also assayed. **b** Same as in (a) but with the VDAC1 variant L10C that is located further inside the pore. **c** Chemical crosslinking of VDAC1 T6C or L10C variants with the amino-selective crosslinker bissulfosuccinimidyl suberate (BS^3). **d** BS^3 crosslinking of VDAC1 in liposomes composed of POPC or POPC/POPG (50:50% (w/w)) lipids. Formation of very large VDAC1 oligomers is enhanced in the presence of a negatively charged lipid surface (PCPG). **e** PM40 modification of the samples shown in (d). Similarly, the negatively charged lipid POPG enhanced the amount of VDAC1 α-helix exposure for both T6C and L10C mutants. Panels **f** and **g** show the effect of the addition of negatively charged CHS into the three types of liposomes (100% POPC or 50:50% (w/w) POPC:POPG or mitochondrial lipid blend containing 40:30:10:10:10% (w/w) POPC:POPE:POPG:DOPS:Cardiolipin) on oligomerization (+BS^3) and VDAC1 α-helix exposure (+PM40). The monomer form of VDAC1 appears as double bands after BS^3 crosslinking: unmodified (upper) and modified with internal crosslink (lower). Each experiment was repeated three times with similar results.

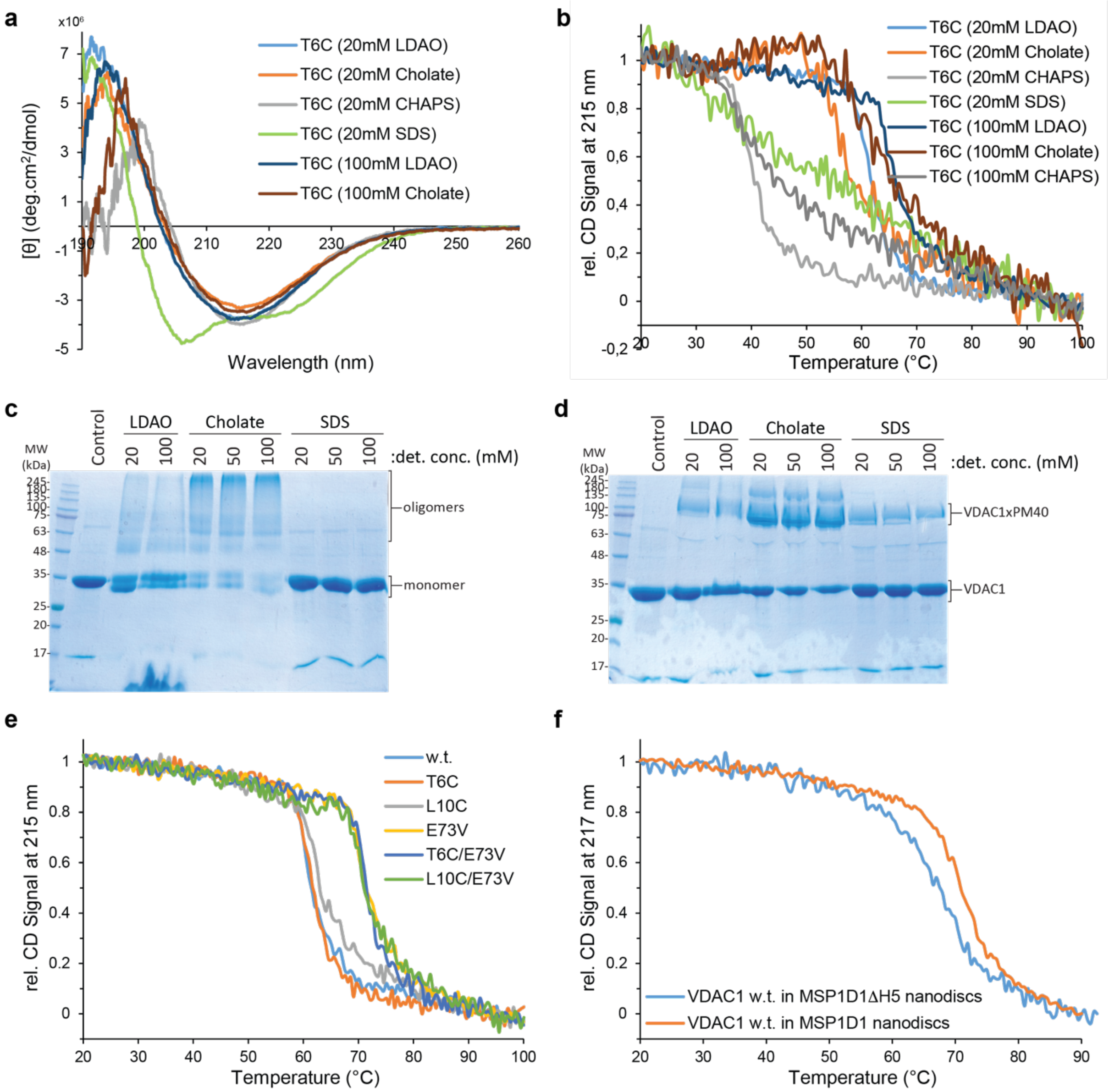

Supplementary Figure 2. **Far-UV CD spectra and CD-detected thermal stability of VDAC1 variants in detergent micelles and nanodiscs.** The experiments were conducted in 1 mm or 0.2 mm pathlength cuvettes. **a** Far-UV CD spectra of VDAC1-T6C at different detergent conditions (20 mM each). While LDAO, cholate and CHAPS resulted in a characteristic β-sheet fold as expected for VDAC1, the presence of SDS changed the secondary structure profile due to its unfolding effects on protein structures. [θ], mean residue weight ellipticity. **b** CD-detected thermal melting curves of the single cysteine mutant in the same detergents. Similarly, while LDAO, cholate and CHAPS resulted in cooperative unfolding curves with different stabilities, this was not the case for SDS. The results in (a) and (b) indicate that all tested detergents except SDS do not interfere with overall fold of VDAC1 at room temperature. **c** The oligomerization of VDAC1 is not dependent on the detergent concentration. **d** Exposure of the N-terminal α-helical segment probed by the PM-40 modification assay is not affected by an increase in the detergent concentration. **e** VDAC1 variants as indicated in the plot in 20 mM LDAO, 20 mM NaPi pH 7.5, 50 mM NaCl, 1 mM DTT. **f** VDAC1 wild-type in MSP1D1 (orange) and MSP1D1ΔH5 (blue) nanodiscs loaded with DMPC:DMPG=3:1 (n/n) lipids in the same buffer. The curves in (b), (e) and (f) were measured at 215 or 217 nm and normalized using the change in the CD signal amplitude between 20°C and 100 or 90°C.

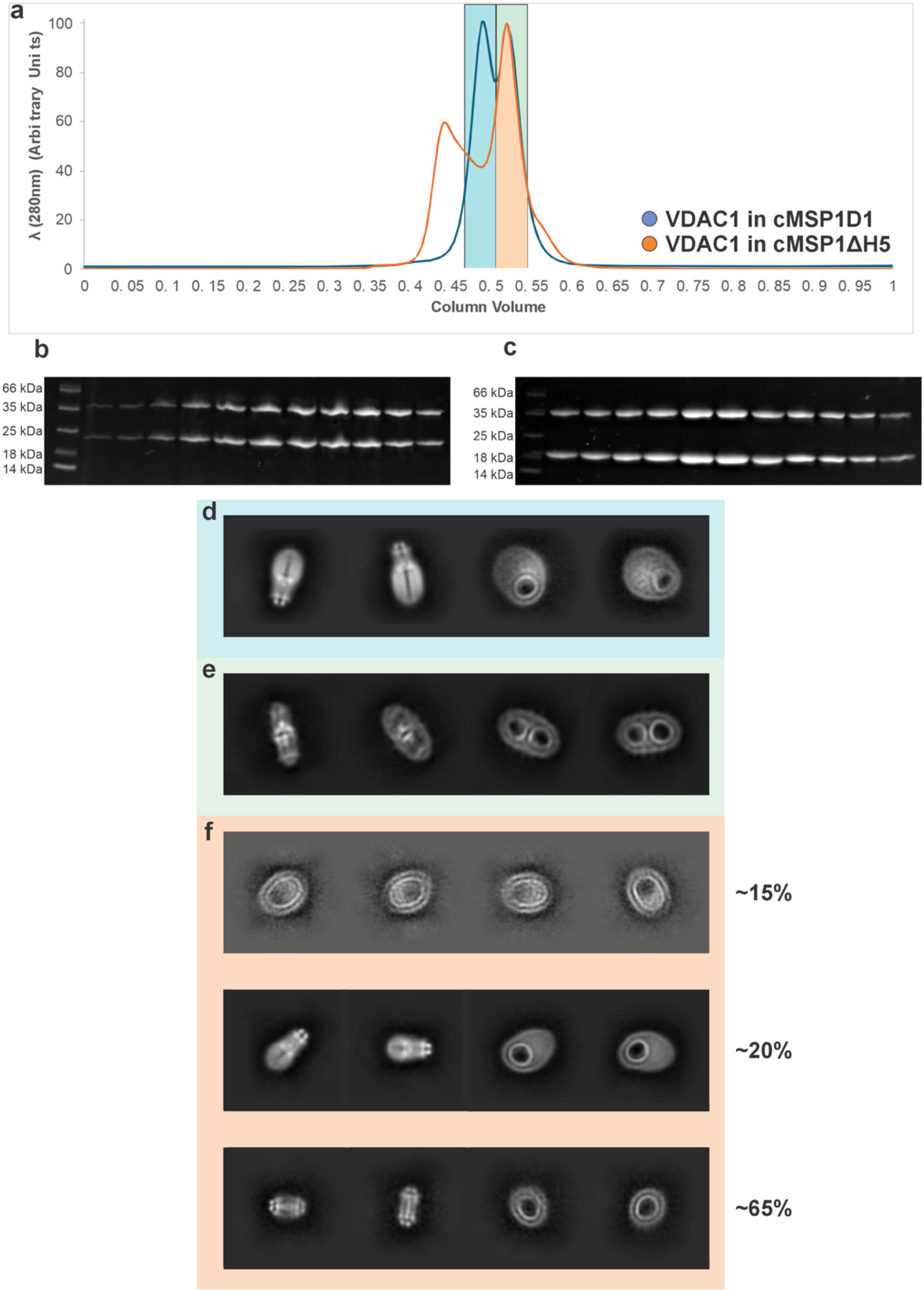

Supplementary Figure 3. **Homogeneity of VDAC1 nanodisc samples and cryo-EM analysis. a** Comparative size exclusion chromatogram of both samples. Fractions collected are highlighted. **b** SDS-PAGE of VDAC1 in cMSP1D1 **c** SDS-PAGE of VDAC1 in cMSP1D1ΔH5. **d-f** Representative 2D class averages of species found in each sample. **d** Large nanodiscs containing a single VDAC1 protomer from the early peak of cMSP1E3D1 VDAC1. **e** Large nanodiscs containing a single VDAC1 dimer from the early peak of cMSP1D1 VDAC1. Notice that monomeric contaminants were found and eliminated in this dataset. **f** Variety of species found in the cMSP1ΔH5 VDAC1 dataset and their relative abundance.

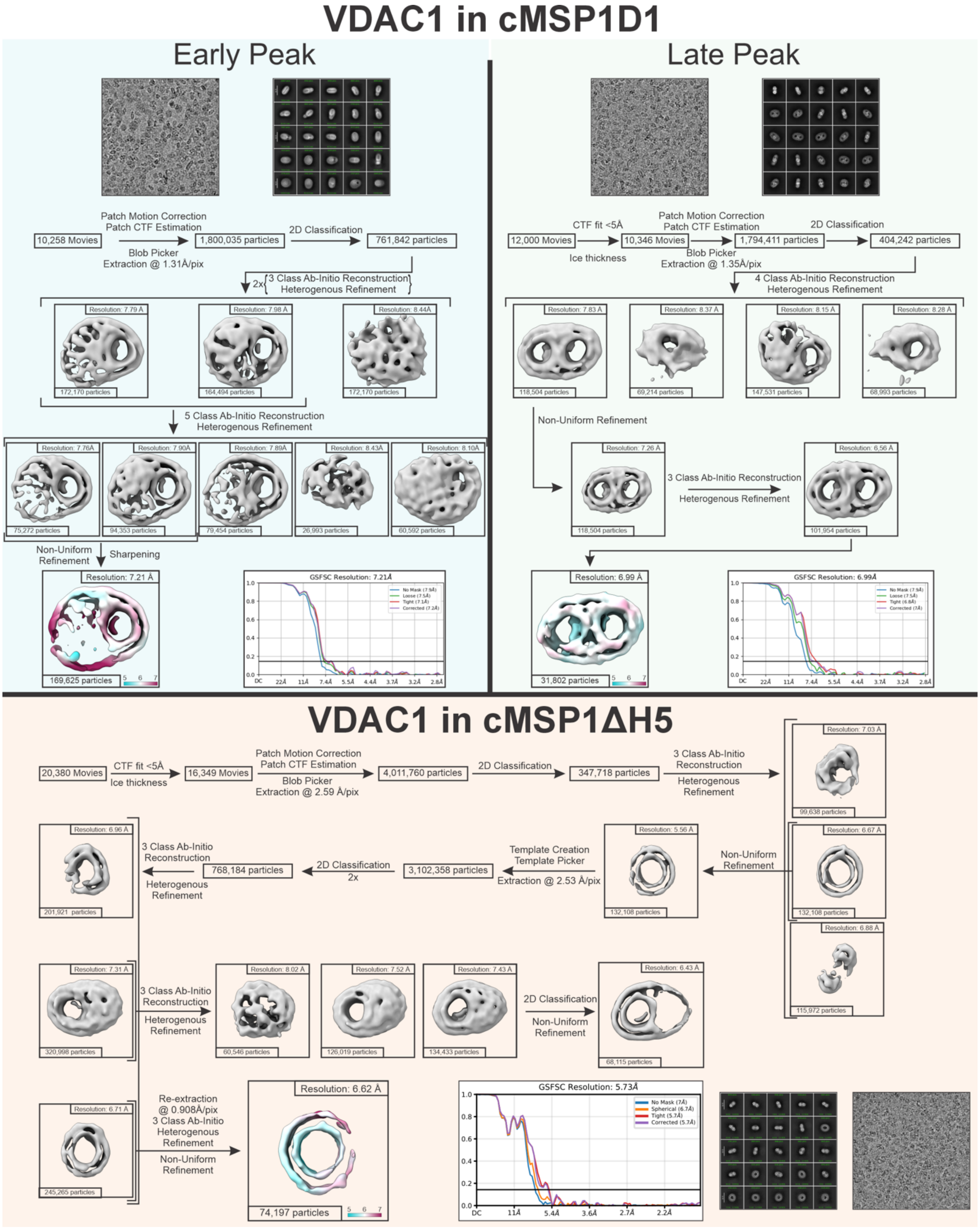

Supplementary Figure 4. **Cryo-EM processing workflow of VDAC1 in nanodiscs of different sizes.** **Top left**: Processing of cMSP1D1 VDAC1 monomers in nanodiscs (peak 1) yielding a final resolution of 7.21 Å. **Top Right**: Processing of cMSP1D1 VDAC1 dimers in nanodiscs (peak 2) giving a final resolution of 6.99 Å. **Bottom**: Processing of cMSP1ΔH5 VDAC1 nanodiscs yielding a resolution of 5.7 Å.

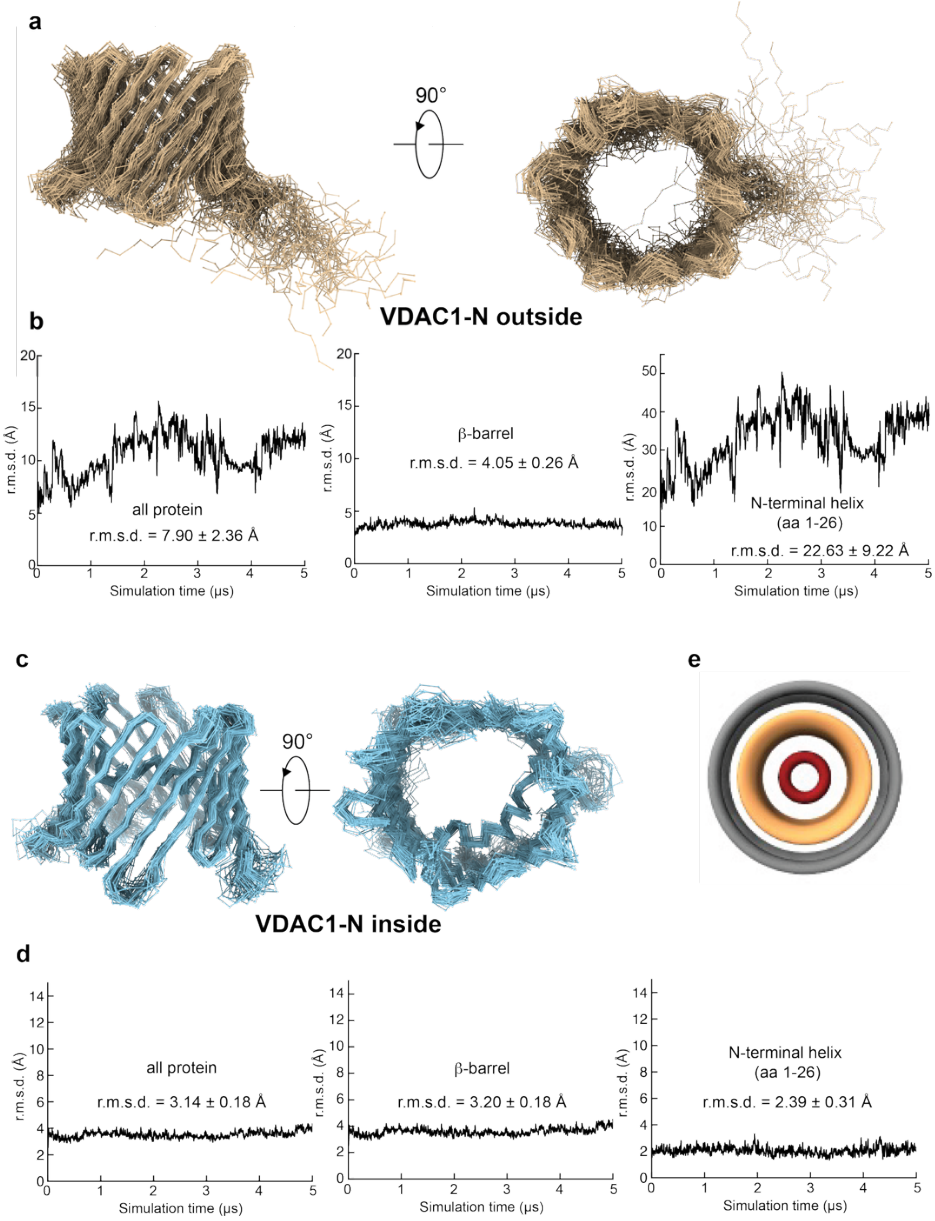

Supplementary Figure 5. **Molecular dynamics (MD) simulations of VDAC1 with the N-terminal helix outside or inside the β-barrel.** **a** Overlay of structural snapshots (every 100 ns) along a 5 µs trajectory of VDAC1 with the N-terminal helix outside the β-barrel in a lipid bilayer composed of POPC and POPG (3:1) at T=310 K. **b** The root mean square deviation (r.m.s.d.) of the atomic coordinates of the entire protein, the β-barrel or just the N-terminal helix obtained from the MD trajectory show that VDAC1 undergoes large conformational fluctuations with an almost unrestricted N-terminal helix (r.m.s.d. up to 40 Å) but also with a less well defined β-barrel. In each case a representative trajectory is shown out of 3 independent MD simulations. The reported values in each panel represent the mean value and the standard deviation with n=3. **c** Same as in (a) but using the canonical human VDAC1 structure (PDB ID: 5XDO) with the N-terminal helix attached to the inside of the β-barrel. **d** The r.m.s.d. values in the latter simulation are much lower with a maximum of ~4 Å and an even lower value for the N-terminal helix (~2 Å), suggesting that the N-terminal helix leads to a marked stabilization of the overall VDAC1 structure. **e** Expected cryo-EM density with the N-terminal helix (red) inside the β-barrel (orange) if the nanodisc particles (grey) are rotationally misaligned, resulting in an additional ring-shaped density inside the pore (see Methods section).

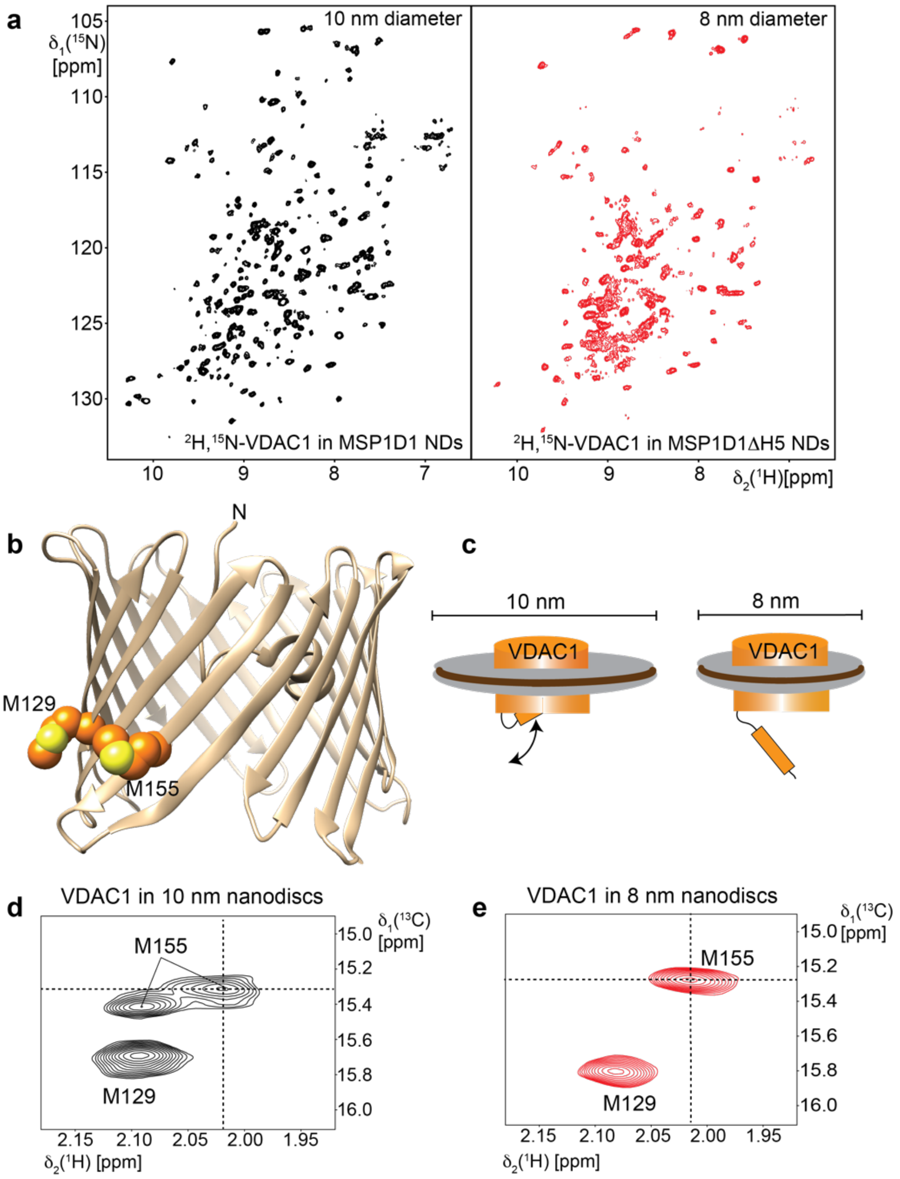

Supplementary Figure 6. **2D-NMR spectra of VDAC1 in nanodiscs of different sizes. a**, left, 2D-[^{15}N,^{1}H]-TROSY spectrum of 200 µM ^{2}H,^{15}N-labeled VDAC1 in MSP1D1 lipid nanodiscs of 10 nm diameter assembled with a DMPC:DMPG=3:1 lipid blend, measured at 800 MHz ^{1}H frequency and at T = 45°C in 20 mM NaPi pH 7.0, 50 mM NaCl, 0.5 mM EDTA, 5 mM DTT buffer. **a**, right, 2D-[^{15}N,^{1}H]-TROSY spectrum of 200 µM ^{2}H,^{15}N-labeled VDAC1 in MSP1D1ΔH5 lipid nanodiscs of 8 nm diameter. The smaller nanodisc leads to marked line broadening of VDAC1 resonances. **b** Structure of VDAC1 with the native methionine residues (M129, M155) labeled. The N-terminal methionine residue is removed during protein expression and is not visible in the spectrum. **c** Cartoon presentation of VDAC1 in 8 and 10 nm nanodiscs, with the N-terminus being exposed in the smaller nanodiscs. **d** 2D-[^{13}C,^{1}H]-HMQC spectrum of Met-ε-^{13}C-labeled VDAC1 in 10 nm nanodiscs. As evident from the peak doubling for M155, VDAC1 exists in two conformational states. **e** same as (d) but with VDAC1 in 8 nm nanodiscs. Only one NMR signal exists for M155 indicating a single conformation. A comparison of the chemical shifts of M155 in both samples, suggests that M155 in 8 nm nanodiscs is present in an exposed conformation and in 10 nm the majority (~60%) is present in the canonical helix-inserted state.

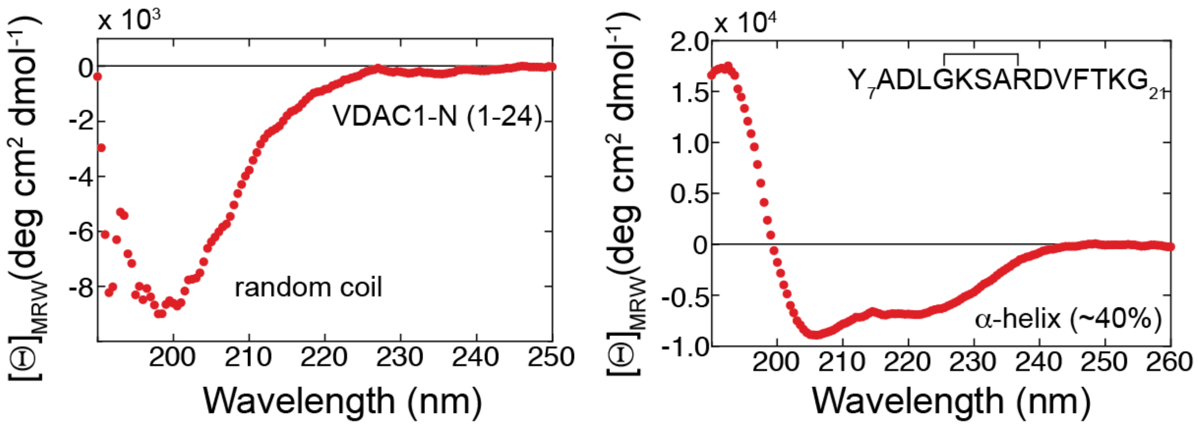

Supplementary Figure 7. **CD spectra of VDAC1-N and st-VDAC1-N.** (Left) CD spectrum of a peptide derived from the VDAC1 N-terminal segment (residues 1-24) indicates a random coil secondary structure. (Right) The CD spectrum of a hydrocarbon stapled VDAC1 peptide (residues 7-21 with the staple between residues 11 and 15) adopts ~40% α-helical secondary structure, as estimated with the program BestSel[2]. Data are represented as mean residue weight (MRW) ellipticity. A 1 mm path length cuvette was used at T = 20°C.

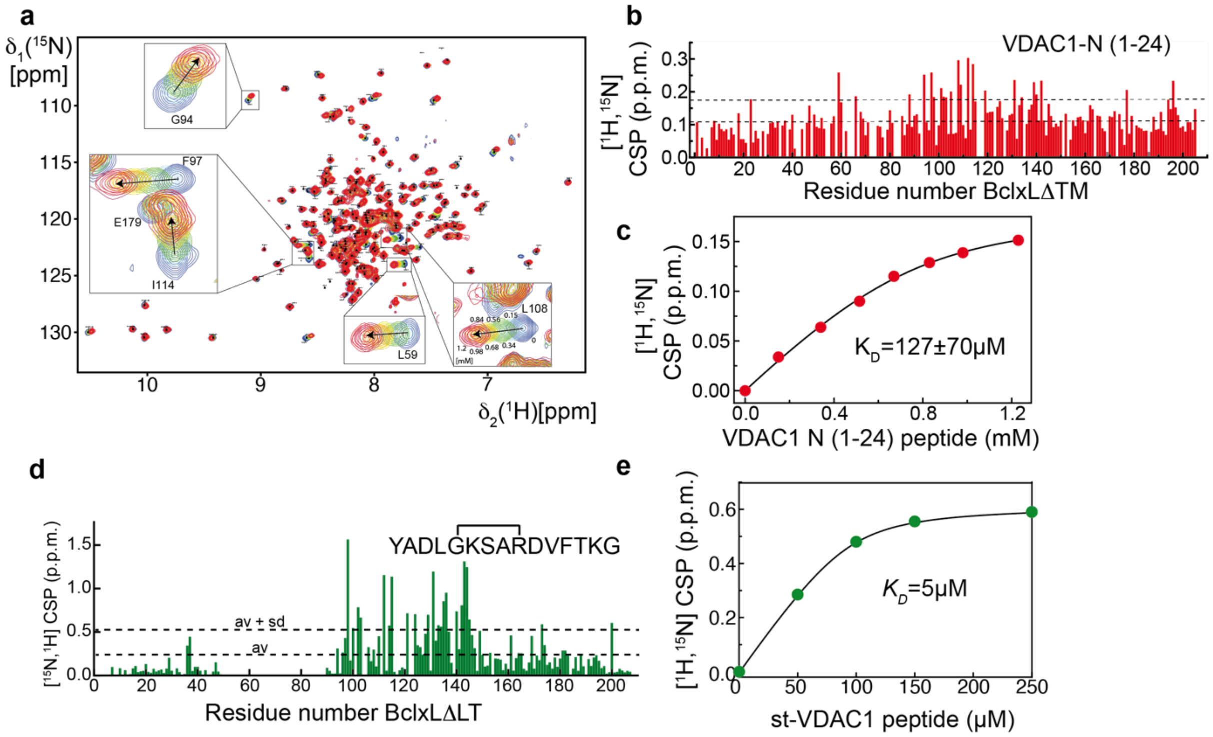

Supplementary Figure 8. **NMR validation of the interaction between VDAC1-N-terminal peptides and BclxL.** **a** 2D-[^{15}N,^{1}H]-TROSY NMR titration with ^{2}H,^{15}N-labeled BclxLΔTM (lacking the transmembrane helix) and the linear VDAC1-N-terminal peptide (residues 1-24). Some affected resonances are shown in the insets. Blue: apo BclxLΔTM, red: 1.2 mM VDAC1-N. **b** Chemical shift perturbation (CSP) values at the highest concentration of VDAC1-N (1.2 mM) for each residue in BclxLΔTM. **c** CSP values in BclxL plotted against the concentration of VDAC1-N, giving rise to a binding isotherm, yielding a K_D value of ~130 µM. **d** CSP pattern of BclxLΔLT (lacking the flexible loop and the transmembrane helix) at a saturating concentration of st-VDAC1-N. The detected CSP amplitude is markedly larger than with the VDAC1-N peptide. **e** Binding isotherm obtained with st-VDAC1-N results in a K_D value of ~5 µM.

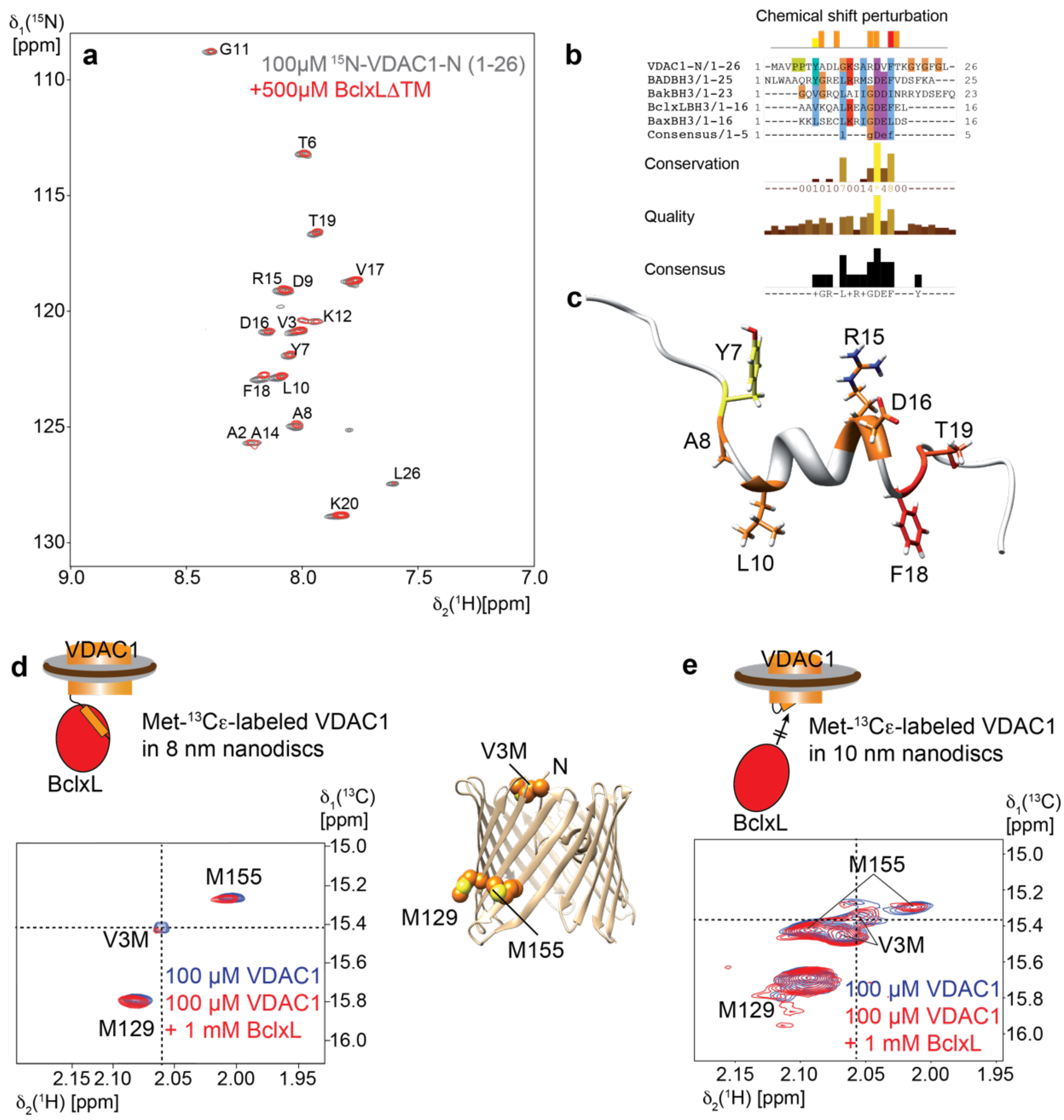

Supplementary Figure 9. **Interaction between VDAC1-N and BclxL at a per-residue resolution obtained by NMR. a** 2D-[^{15}N,^{1}H]-TROSY spectra of ^{15}N-labeled VDAC1-N (1-26)[3] and after the addition of a 5-fold amount of BclxL. **b** Chemical shift perturbations (CSPs) were observed for the indicated positions with yellow, orange and red representing weak, medium and strong effects, respectively. Below the CSPs is a multiple sequence alignment of VDAC1-N with BH3 peptides from various Bcl2 proteins, indicating a low level of conservation. **c** CSPs mapped onto the structure of VDAC1-N taken from the NMR structure of full-length VDAC1 in detergent micelles[4]. **d** 2D-[^{13}C,^{1}H]-HMQC spectrum of Met-ε-^{13}C-labeled VDAC1 in 8 nm lipid nanodiscs alone (blue) and in presence of a 10-fold molar excess of BclxL (red). The NMR signal for Met3 is mostly affected by the addition of BclxL. Methionine chemical shift assignments were obtained by mutagenesis. **e** same as in (d) but with VDAC in 10 nm nanodiscs. There is peak doubling for Met3 and Met115, indicative of two conformational states. For Met3, the minor peak (crosshair) is affected by the addition of BclxL. A comparison with the spectrum in (d) suggests that this peak represents the exposed conformation of the VDAC1 N-terminus. The main conformational state of each methionine residue (M155 and M3) has a population of 60-70%, as determined by the peak intensities of each resonance. This feature indicates a correlation between the two sites.

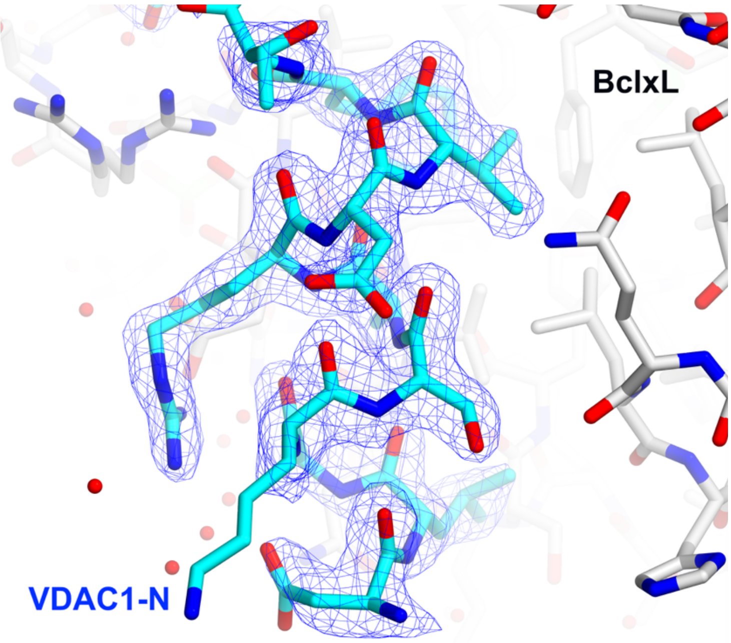

Supplementary Figure 10. **2Fo-Fc electron density map contoured at 1σ for the VDAC1-N peptide (cyan) bound to BclxL (gray).**

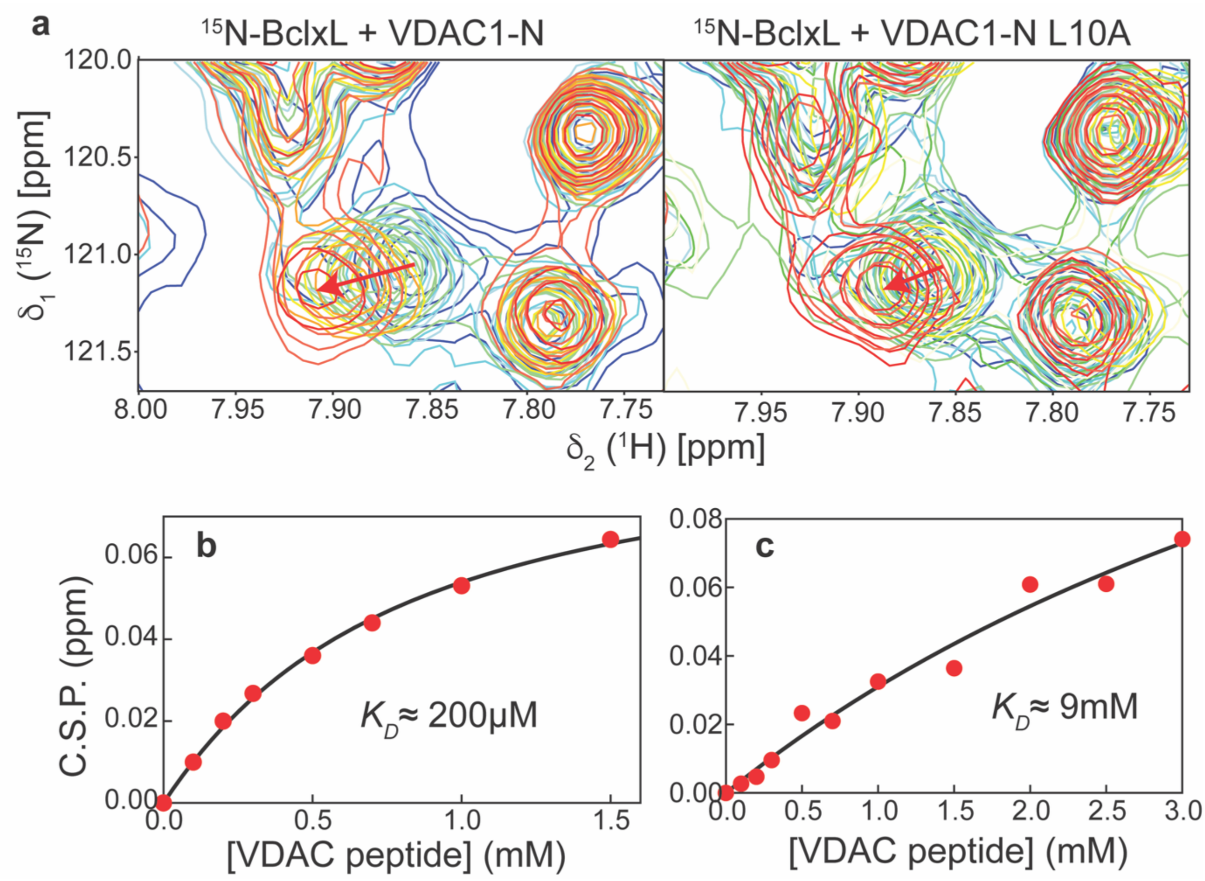

Supplementary Figure 11. **Alanine scan of the VDAC1-N peptide and its interaction with BclxL. a** NMR spectral overlays of the titration steps with the wild-type (left) or the L10A peptide (right) at increasing concentrations (dark blue: apo, red: final concentration, 1.5 mM for the wild-type peptide and 3 mM for the L10A peptide). **b** NMR-derived binding isotherm with the wild-type VDAC1-N peptide. **c** same as (b) but with the L10A VDAC1-N peptide.

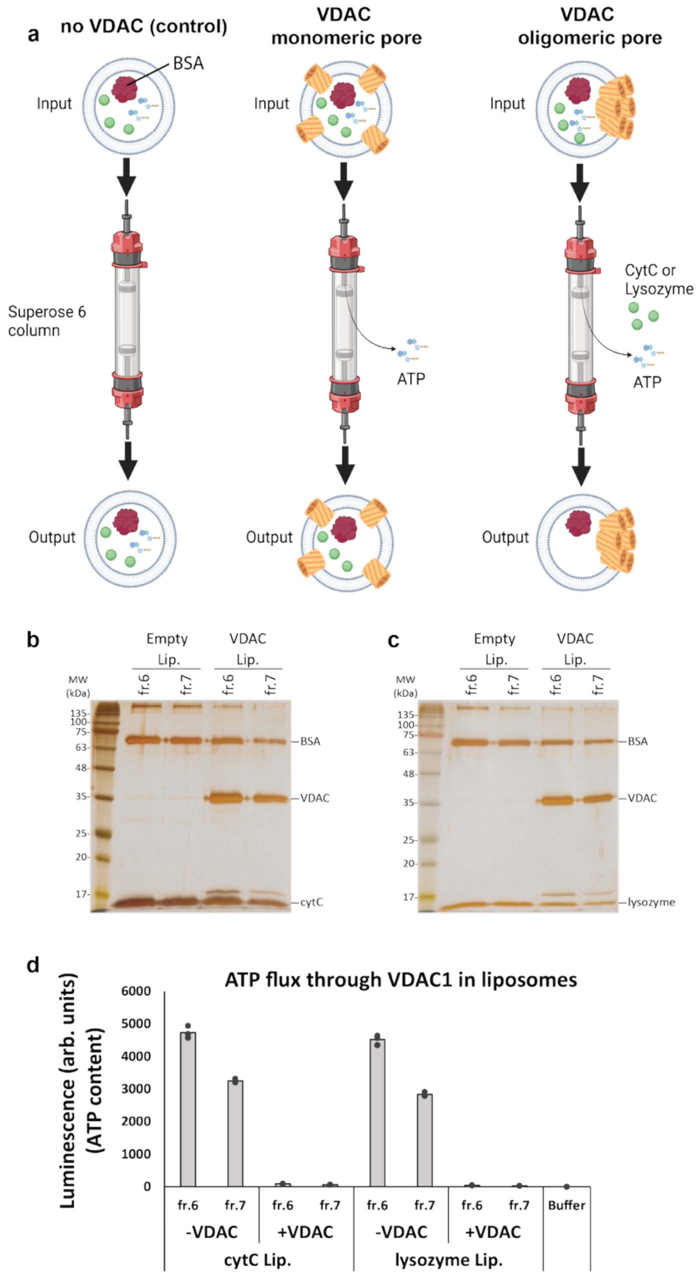

Supplementary Figure 12. **VDAC1 in liposomes is permeable for ATP but not for small proteins. a** Schematic of the size exclusion assay to probe ATP or cytochrome C/lysozyme translocation across VDAC1. **b** Liposomes loaded with bovine serum albumin (BSA) and cytochrome C (cytC) with or without VDAC1 were injected on a Superose 6 size exclusion chromatography column (Cytvia). If proteins inside the liposomes are able exit the liposomes the corresponding band on the SDS-PAGE should be markedly weakened or be completely lost. The ~70 kDa BSA protein serves as a reference since it is assumed to be too large to exit through a VDAC1 pore. For cytC, no clear reduction in the band intensity could be observed. **c** same as in (b) but with lysozyme instead of cytC, showing the same result, i.e. no translocation of the protein across VDAC1. **d** To probe the functionality of VDAC1 and the existence of a pore that is large enough to allow for the transition of metabolites, the amount of ATP in empty and VDAC1-containing liposomes was monitored. In contrast to both proteins in (b) and (c) ATP was completely removed in VDAC1 proteoliposomes but not in liposomes without VDAC1, indicating its efficient translocation across VDAC1. Data points are three technical replicates (n=3). Bar height: mean value. Panel a was created in BioRender: Hagn, F. (2025) https://BioRender.com/a9q1pjxwas

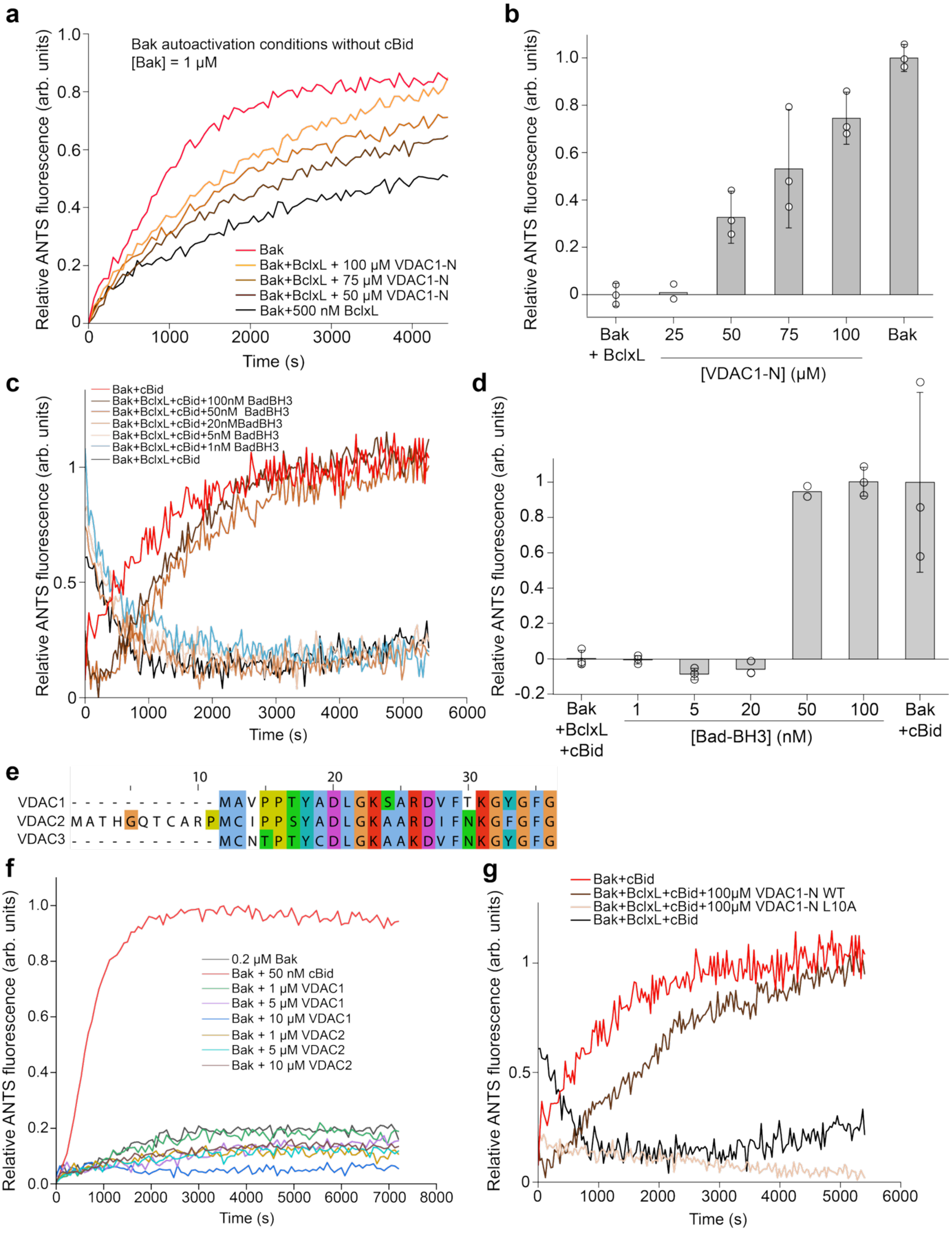

Supplementary Figure 13. **VDAC-N peptides cannot directly activate pore formation of Bak. a** BakΔTM (1 μM concentration, red) shows autoactivation which can be partially inhibited by the addition of 500 nM BclxLΔTM (black). The addition of stVDAC1-N (50 - 100 μM) induces Bak pore formation in the presence of BclxL in a concentration-dependent manner (brown to yellow colors). **b** Fluorescence intensity taken from panel (a) averaged between 4000 and 5000 s. The standard deviation (error bars) was calculated from at least two technical replicates (circles). **c** Pore formation of cBid-activated BakΔTM (40 nM cBid: 50 nM Bak, red) is inhibited by the addition of BclxLΔTM (50 mM,

black). The addition of the prototype sensitizer Bad-BH3 peptide (1-100 nM) induces Bak pore formation in the presence of BclxL at equimolar concentrations of peptide to BclxL (brown). **d** Fluorescence intensities from panel (c) averaged between 5000 and 5500 s. The standard deviation (error bars) was calculated from at least two technical replicates (circles). **e** Multiple sequence alignment of human VDAC1, VDAC2 and VDAC3 N-terminal regions shows an almost identical sequence for VDAC1 and VDAC3 and a 10 amino acid residue N-terminal extension in VDAC2. Coloring was done by the Clustral method in Jalview[5]. **f** Normalized fluorescence intensity of ANTS in a liposome pore forming assay [6] with 200 nM Bak and different concentrations of VDAC1-N and VDAC2-N, as well as cBid as a positive control. Bak pore formation requires activation by BH3-only proteins, as seen for cBid (50 nM, red curve). In comparison, up to 10 µM of either GB1-VDAC1-N (blue) or GB1-VDAC2-N (green) peptides were unable to activate Bak. **g** Pore formation of cBid (40 nM)-activated BakΔTM (50 nM, red) is inhibited by the addition of BclxLΔTM (50 nM, black). The addition of linear VDAC1-N WT peptide (100 µM, brown, see Tab. S4 for sequence information) induces Bak pore formation in the presence of BclxL, whereas the addition of mutated linear VDAC1-N L10A peptide (100 µM, beige, see Supplementary Table 4 for sequence information) is not capable of inducing pore formation.[5]

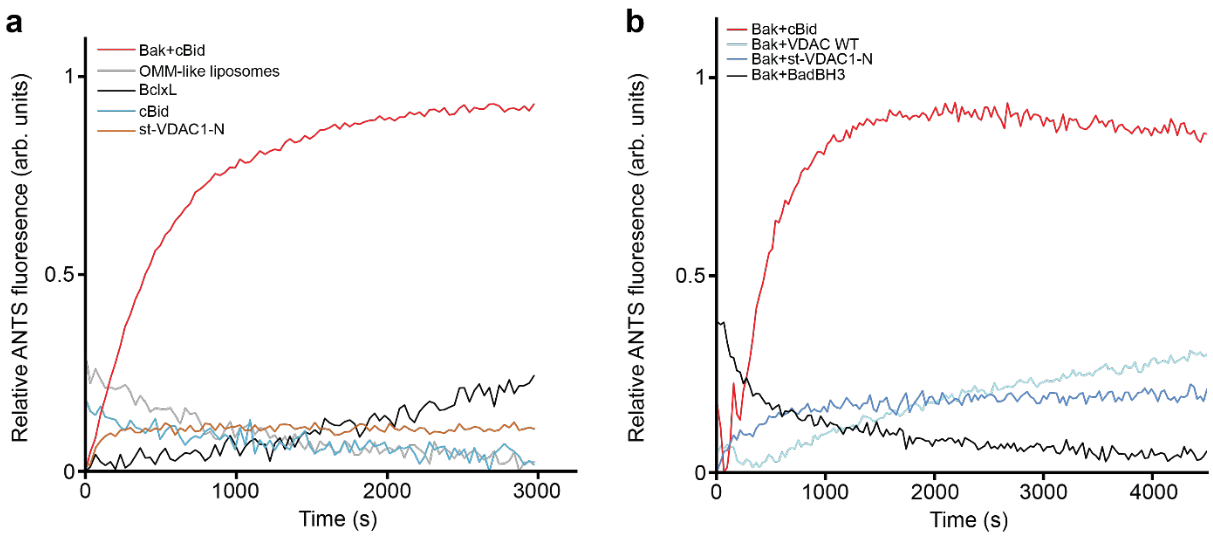

Supplementary Figure 14. **Controls of the liposome pore forming assay. a** Control experiments with single components that have been used in the pore forming assay: 50 nM Bak+40 nM cBid, 40 nM cBid, 70 µM stVDAC1-N. Only activated Bak in presence of cBid forms pores. **b** Bak alone at 50 nM concentration does not form pores but needs activation by cBid (40 nM). Addition of 100 nM of the known sensitizer BH3 protein Bad, 100 µM stVDAC1-N or 1 mM linear VDAC1-N do not directly activate Bak.

Supplementary References

1 Villinger, S. *et al.* Functional dynamics in the voltage-dependent anion channel. *Proc Natl Acad Sci U S A* **107**, 22546-22551 (2010). https://doi.org:10.1073/pnas.1012310108

2 Micsonai, A. *et al.* Accurate secondary structure prediction and fold recognition for circular dichroism spectroscopy. *Proc Natl Acad Sci U S A* **112**, E3095-3103 (2015). https://doi.org:10.1073/pnas.1500851112

3 Reif, M. M., Fischer, M., Fredriksson, K., Hagn, F. & Zacharias, M. The N-Terminal Segment of the Voltage-Dependent Anion Channel: A Possible Membrane-Bound Intermediate in Pore Unbinding. *J Mol Biol* **431**, 223-243 (2019). https://doi.org:10.1016/j.jmb.2018.09.015

4 Hiller, S. *et al.* Solution structure of the integral human membrane protein VDAC-1 in detergent micelles. *Science* **321**, 1206-1210 (2008). https://doi.org:10.1126/science.1161302

5 Waterhouse, A. M., Procter, J. B., Martin, D. M., Clamp, M. & Barton, G. J. Jalview Version 2--a multiple sequence alignment editor and analysis workbench. *Bioinformatics* **25**, 1189-1191 (2009). https://doi.org:10.1093/bioinformatics/btp033

6 Kale, J., Chi, X., Leber, B. & Andrews, D. Examining the molecular mechanism of bcl-2 family proteins at membranes by fluorescence spectroscopy. *Methods Enzymol* **544**, 1-23 (2014). https://doi.org:10.1016/B978-0-12-417158-9.00001-7
