# Supplementary material for: Structural basis of apoptosis induction by the mitochondrial voltage-dependent anion channel
Source: Nat Commun. 2025 Oct 27;16:9481. doi: 10.1038/s41467-025-65363-1 (PMC12559223; doi:10.1038/s41467-025-65363-1)
Supplement: Supplementary file 2 — Reporting Summary [file 41467_2025_65363_MOESM2_ESM.pdf]

nature portfolio

Corresponding author(s): Franz Hagn

Last updated by author(s): Sep 24, 2025

Reporting Summary

Nature Portfolio wishes to improve the reproducibility of the work that we publish. This form provides structure for consistency and transparency in reporting. For further information on Nature Portfolio policies, see our Editorial Policies and the Editorial Policy Checklist.

Statistics

For all statistical analyses, confirm that the following items are present in the figure legend, table legend, main text, or Methods section.

n/a	Confirmed	
☐	☒	The exact sample size (n) for each experimental group/condition, given as a discrete number and unit of measurement
☐	☒	A statement on whether measurements were taken from distinct samples or whether the same sample was measured repeatedly
☒	☐	The statistical test(s) used AND whether they are one- or two-sided<br>*Only common tests should be described solely by name; describe more complex techniques in the Methods section.*
☒	☐	A description of all covariates tested
☒	☐	A description of any assumptions or corrections, such as tests of normality and adjustment for multiple comparisons
☐	☒	A full description of the statistical parameters including central tendency (e.g. means) or other basic estimates (e.g. regression coefficient) AND variation (e.g. standard deviation) or associated estimates of uncertainty (e.g. confidence intervals)
☒	☐	For null hypothesis testing, the test statistic (e.g. F, t, r) with confidence intervals, effect sizes, degrees of freedom and P value noted<br>*Give P values as exact values whenever suitable.*
☒	☐	For Bayesian analysis, information on the choice of priors and Markov chain Monte Carlo settings
☒	☐	For hierarchical and complex designs, identification of the appropriate level for tests and full reporting of outcomes
☒	☐	Estimates of effect sizes (e.g. Cohen's d, Pearson's r), indicating how they were calculated

Our web collection on statistics for biologists contains articles on many of the points above.

Software and code

Policy information about availability of computer code

Data collection	Unicorn7 (Cytiva), SoftMax Pro 7 Software (Molecular Devices plate reader), Spectra Manager (Jasco), PEAQ-ITC Control Software v1.41, Topspin v4.0 (Bruker), EPU software (Thermo Fisher Scientific), NAMD v.12, CUDA-enhanced version of GROMACS 2025,
Data analysis	Unicorn7 (Cytiva), MicroCal PEAQ-ITC Analysis Software v1.41, Topspin v4.0 (Bruker), XDS (v. 01.10.14), SCALA (v. 01.10.14), TRUNCATE (v. 8.0.019), PHASER, v. 2.8.3, COOT (v. 0.9.8.93), PROCHECK (v. 3.5.4.) and MolProbity (v. 4.02b-467), REFMAC5 (v. 5.8.0158), NMRFAM-SPARKY 1.3, CryoSPARC Live, CryoSPARC (v3.1.0-v4.5.1), GROMACS 2025, VMD v.1.9.4, ChimeraX-1.9, OriginPro (OriginLab)

For manuscripts utilizing custom algorithms or software that are central to the research but not yet described in published literature, software must be made available to editors and reviewers. We strongly encourage code deposition in a community repository (e.g. GitHub). See the Nature Portfolio guidelines for submitting code & software for further information.

Data

Policy information about availability of data

All manuscripts must include a data availability statement. This statement should provide the following information, where applicable:

- Accession codes, unique identifiers, or web links for publicly available datasets
- A description of any restrictions on data availability
- For clinical datasets or third party data, please ensure that the statement adheres to our policy

The atomic coordinates have been deposited at the Protein Data Bank (PDB) under accession code 9HPS [https://doi.org/10.2210/pdb9HPS/pdb] (X-ray structure of BclxLLT bound to the VDAC1-N-terminal-helix). This structure was solved by molecular replacement using PDB code 1MAZ101[https://doi.org/10.2210/

pdb1maz/pdb](BclxL apo structure). The herein determined cryo-EM maps have been deposited to the Electron Microscopy Data Bank (EMDB) under the accession codes EMD-55061 [https://www.ebi.ac.uk/emdb/EMD-55061]. (VDAC1 monomer in MSP1D1 nanodiscs), EMD-55062 [https://www.ebi.ac.uk/emdb/EMD-55062] (VDAC1 dimer in MSP1D1 nanodiscs), and EMD-55094 [https://www.ebi.ac.uk/emdb/EMD-55094] (VDAC1 monomer in MSP1D1H5 nanodiscs), respectively. Source data generated in this study are provided in the Supplementary Information/Source Data. MD input files and calculated trajectories for VDAC1 with different positions of the N-terminal helix (5 µs each), as well as the energy-minimized structural model of the VDAC1-Bclxl complex generated in this study are available in the FigShare repository [https://figshare.com/s/dce14b2c4b97eff221f9].

Research involving human participants, their data, or biological material

Policy information about studies with human participants or human data. See also policy information about sex, gender (identity/presentation), and sexual orientation and race, ethnicity and racism.

Reporting on sex and gender	n/a
Reporting on race, ethnicity, or other socially relevant groupings	n/a
Population characteristics	n/a
Recruitment	n/a
Ethics oversight	n/a

Note that full information on the approval of the study protocol must also be provided in the manuscript.

Field-specific reporting

Please select the one below that is the best fit for your research. If you are not sure, read the appropriate sections before making your selection.

- [x] Life sciences
- [ ] Behavioural & social sciences
- [ ] Ecological, evolutionary & environmental sciences

For a reference copy of the document with all sections, see nature.com/documents/nr-reporting-summary-flat.pdf

Life sciences study design

All studies must disclose on these points even when the disclosure is negative.

Sample size	1
Data exclusions	none
Replication	n=3
Randomization	none
Blinding	none

Reporting for specific materials, systems and methods

We require information from authors about some types of materials, experimental systems and methods used in many studies. Here, indicate whether each material, system or method listed is relevant to your study. If you are not sure if a list item applies to your research, read the appropriate section before selecting a response.

Materials & experimental systems

n/a	Involved in the study	
x		Antibodies
x		Eukaryotic cell lines
x		Palaeontology and archaeology
x		Animals and other organisms
x		Clinical data
x		Dual use research of concern
x		Plants

Methods

n/a	Involved in the study	
x		ChIP-seq
x		Flow cytometry
x		MRI-based neuroimaging

Plants

Seed stocks	n/a
Novel plant genotypes	n/a
Authentication	n/a
